# Structural Posterior Fossa Malformations: MR Imaging and Neurodevelopmental Outcome

**DOI:** 10.3390/diagnostics15151945

**Published:** 2025-08-03

**Authors:** Jorden Halevy, Hadar Doitch Amdurski, Michal Gafner, Shalev Fried, Tomer Ziv-Baran, Eldad Katorza

**Affiliations:** 1 Department of Obstetrics and Gynecology, Chaim Sheba Medical Center, Tel-Hashomer, Derech Sheba 2, Ramat Gan 5262000, Israel; eldad.katorza@sheba.health.gov.il; 2 Department of Pediatrics, Dana-Dwek Children’s Hospital, Tel Aviv Sourasky Medical Center, 6 Weizmann Street, Tel Aviv 6423906, Israel; 3 Faculty of Health Sciences and Medicine, Tel Aviv University, Tel Aviv 6997801, Israel; michalgurevitch@gmail.com (M.G.);; 4 Department of Pediatrics B, Schneider Children’s Medical Center of Israel, 14 Kaplan Street, Petach Tikva 4920235, Israel; 5 The Goldschleger Eye Institute, Chaim Sheba Medical Center, Tel-Hashomer, Derech Sheba 2, Ramat Gan 5262000, Israel; 6 Gertner Institute for Epidemiology & Health Policy Research, Chaim Sheba Medical Center, Tel-Hashomer, Derech Sheba 2, Ramat Gan 5262000, Israel

**Keywords:** magnetic resonance imaging, brain, child development, fetus, nervous system malformations, cranial fossa, posterior

## Abstract

**Objectives**: The increasing use of fetal MRI has increased the diagnosis of posterior fossa malformations, yet the long-term neurodevelopmental outcomes of affected fetuses remain unclear. This study aims to examine the long-term neurodevelopmental outcomes of fetuses with structural posterior fossa malformation diagnosed on fetal MRI. **Methods**: A historical cohort study was conducted at a single tertiary referral center, including fetuses diagnosed with structural posterior fossa malformations and apparently healthy fetuses who underwent fetal brain MRI between 2011 and 2019. Maternal, pregnancy, and newborn characteristics were compared between groups, alongside long-term neurodevelopmental outcomes using the Vineland Adaptive Behavior Scales II (VABS-II) questionnaire. This included an extensive assessment of malformation types, additional structural, genetic, or neurodevelopmental anomalies, and outcomes. **Results**: A total of 126 fetuses met the inclusion criteria, of which 70 were apparently healthy fetuses, and 56 had structural posterior fossa malformations. Among the latter, 18 pregnancies were terminated, 4 resulted in neonatal death, and 11 were lost to follow-up. No significant differences were found in the overall neurodevelopmental outcomes between fetuses with structural posterior fossa malformation (93.4 ± 19.0) and apparently healthy fetuses (99.8 ± 13.8). Motor skills scores were lower among fetuses with structural posterior fossa malformations (87.7 ± 16.5 vs. 99.3 ± 17.2, *p* = 0.01) but remained within the normal range. **Conclusion**: Fetuses with structural posterior fossa malformations may exhibit normal long-term neurodevelopmental outcomes if no additional anomalies are detected during thorough prenatal screening that includes proper sonographic, biochemical and genetic screening, as well as fetal MRI. Further research with larger cohorts and longer-term assessments is recommended to validate these findings and support clinical decision-making.

## 1. Introduction

Posterior fossa malformations encompass a heterogeneous spectrum of conditions characterized by abnormal development of the sub-tentorial area [1]. Posterior fossa development begins around the 5th week of gestation, following the formation of the primary neural tube and its subsequent division into the three primary vesicles that form the brain. The rhombencephalon, the vesicle forming the hindbrain, further divides into secondary vesicles—the metencephalon, forming the pons and cerebellum, and the myelencephalon, forming the medulla [2]. Between weeks 6 and 12, ossification of posterior fossa bones, such as the occipital bone and clivus, occurs, forming from paraxial mesoderm and neural crest cells [3]. This intricate process of embryogenesis is vulnerable to disruptions from genetic mutations or errors in developmental timing or patterning [2,4]. Such failures can lead to malformations like Dandy–Walker malformation (due to lack of plica choroidalis involution, leading to an enlarged fourth ventricle that disrupts the proper formation of the vermis and cerebellar hemispheres) [2] or Chiari malformations (resulting from an underdeveloped occipital bone) [3].

Classification of posterior fossa malformations is controversial due to the variable genetic, embryologic, and morphologic bases for these malformations. One suggested classification of these malformations divides them into two groups. The first group comprises hindbrain malformations, which can be further divided into cerebellar and/or vermian malformations and cystic malformations (Blake’s pouch, Dandy–Walker malformation). The second group consists of cranial vault malformations (Chiari malformations) [5]. When suspicion for posterior fossa malformation arises during routine prenatal ultrasonography (US), further examination is needed, including magnetic resonance imaging (MRI) and genetic analysis. Outcomes of these malformations vary considerably according to the type of anomaly [1], with the possibility of major neurological deficits among some of these malformations.

While fetal evaluation during pregnancy is commonly performed by US, MRI is increasingly utilized to evaluate the fetal brain, particularly when an anomaly is detected via US, or in cases of increased risk for neurodevelopmental abnormalities [6]. In some aspects, such as biometry, gyration/sulcation, and characterization of the cerebral parenchyma, MRI is superior to US, especially for evaluating the posterior fossa in late pregnancy [7]. In a subgroup analysis of the MERIDIAN cohort by Griffiths et al., involving 306 fetuses with ventriculomegaly, US failed to detect additional brain anomalies in 31 cases, whereas MRI correctly diagnosed 27 of these missed cases. Thus, in this analysis, US showed 89.9% diagnostic accuracy, compared to 98.7% for MRI [8]. That being said, these two imaging modalities are considered complementary rather than competitive [9].

The increasing utilization of fetal MRI has led to an increase in prenatal detection and diagnostic accuracy of posterior fossa malformations in recent years. This increase in detection is accompanied by a growing discrepancy with the ability to predict neurodevelopmental prognosis, as neurodevelopmental and functional outcomes of children with posterior fossa malformations diagnosed by fetal MRI remain poorly defined [10,11,12,13]. With the current literature being frequently inconsistent regarding these outcomes, clinicians may struggle with counseling families about termination of pregnancy or about expected future struggles [14]. Furthermore, the spectrum of disability displayed in research is broad, ranging from normal development to profound disability for a given malformation [11].

This study will evaluate the immediate postnatal outcome and long-term neurodevelopmental outcome of structural posterior fossa malformations characterized by fetal MRI, comparing these outcomes with those of an apparently healthy fetus with no detected pathology on fetal MRI.

With this study, we aim to address the lacking data, providing further information regarding the prognosis and neurodevelopmental outcomes of structural posterior fossa malformations, and aiding in parental counseling and clinical decision-making [10,14].

## 2. Materials and Methods

This study was approved by the local institutional review board. Informed consent was obtained from all participants: a letter of explanation was mailed to subjects, and oral consent for participation was obtained during the telephone call when conducting the VABS-II interview.

This is a historical cohort study during which fetal MRI scans were obtained between 2011 and 2019 in a single tertiary medical center. Data regarding medical history, sonography and MRI, perinatal history, and medical follow-up were retrieved from patients’ medical records.

### 2.1. Study Population

The study population included two groups: fetuses with posterior fossa malformations observed in fetal MRI and fetuses with no pathology on fetal MRI (‘apparently healthy fetuses’, as will be further described). The posterior fossa malformation group consisted of 56 fetuses with structural posterior fossa malformation diagnosed with fetal MRI. Indications for fetal MRI included a previous child with prenatal neurologic findings, a previous abnormal pregnancy, fetal ultrasound screening with abnormal cranial findings, and suspicion of cytomegalovirus infection ruled out by amnio-polymerase chain reaction. The group of apparently healthy fetuses was a cohort of 70 fetuses that underwent fetal MRI in our institution, for the same indications as described for the first group, and their MRI was interpreted as without pathology. This cohort included solely fetuses whose parents agreed to participate in the VABS-II questionnaire; therefore, adherence among this group cannot be assessed. Inclusion and exclusion criteria for this research are further described in Table 1.

This study did not engage with mega cisterna magna (MCM) or arachnoid cyst, as these anomalies are of functional rather than structural origin. The structural malformations included in this study were Dandy–Walker malformation (DWM), Blake’s pouch cyst, cerebellar hypoplasia and agenesis, pontocerebellar hypoplasia, Joubert syndrome, Chiari II malformation, rhombencephalosynapsis, and partial or complete vermian agenesis.

### 2.2. MR Imaging

Fetal MRI was performed and evaluated by an interdisciplinary team composed of an obstetrician specializing in fetal US and MRI, an expert MRI neuroradiologist, and an experienced pediatric neurologist in group analysis. A 1.5T MR imaging system (Optima 1.5T; GE Healthcare, Milwaukee, Wisconsin) was used. Single-shot fast spin-echo T2 weighted sequences in 3 orthogonal planes were used, with a section thickness of 3–4 mm, no gap, and a flexible coil (8-channel cardiac coil). The field of view (FOV) was determined by fetal head size: 24 cm for smaller fetuses and up to 30 cm for larger ones. Other parameters included a matrix of 320/224, a TE of 90 ms, and a TR of 1298 ms. The fast spoiled gradient-echo T1 sequence was performed only in the axial plane, with a larger FOV of 40 cm, 4 mm section thickness, 0.5-mm gap, a TR of 160 ms, and a TE of 2.3 ms.

### 2.3. Neurodevelopmental Outcome

For evaluating the neurodevelopmental outcomes of the fetuses, the Vineland-II Adaptive Behavior Scales (VABS-II) [15] were utilized. These scales measure adaptive behavior and are considered a reliable and valid assessment tool for neurodevelopment [15,16,17]. The VABS-II is a structured questionnaire measuring adaptive behavior of an individual’s performance in four major domains: communication, daily living skills, socialization, and motor skills, resulting in a score with a mean of 100 ± 15. Scores are considered abnormal if the standard score is <85 in any domain.

The VABS-II version administered was the local published form of the country where this study was conducted. As this published version has no available norms for the full age range, the American norms, which should conform to our data as well, were utilized for this study [18]. The questionnaires were collected through telephonic interviews with mothers, conducted by medical students who were guided by a professional with experience in administering this questionnaire. The students were unaware of the child’s diagnosis. Agreement to participate was requested prior to conducting the questionnaire.

In addition, records of neurologic clinical follow-up were collected retrospectively.

### 2.4. Statistical Analysis

The distribution of continuous variables was evaluated using the Kolmogorov–Smirnov test. Normally distributed continuous variables were reported as means and standard deviations, while other continuous variables were summarized as medians and interquartile ranges. Categorical variables were described as frequencies and percentages. An independent sample *T*-test was used for normally distributed continuous variables, while the Mann–Whitney test was the test of choice for continuous variables that were not normally distributed. Categorical variables were compared between groups using the χ^2^ test or Fisher’s exact test, if the test assumptions for the χ^2^ test were not met. All statistical tests were two-tailed, and *p* < 0.05 was considered statistically significant. SPSS Statistics for Windows, version 25, IBM Corp. (2017), Armonk, NY, USA, was used for all statistical analyses.

## 3. Results

**Figure 1:**
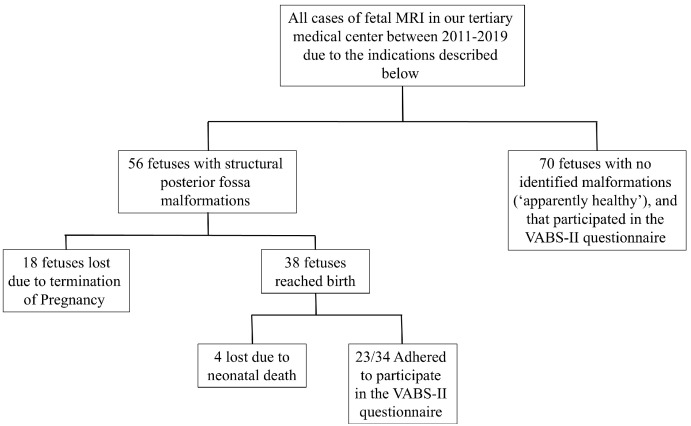
Flowchart of the study design. MRI—magnetic resonance imaging, VABS-II—Vineland Adaptive Behavior Scales II.

The study population included 126 fetuses;56 fetuses with structural posterior fossa malformations observed in fetal MRI that met the inclusion criteria stated earlier, and a cohort of 70 apparently healthy fetuses that underwent fetal MRI in our institute, as described in the Materials and Methods Section above. A total of 22 fetuses with structural posterior fossa malformations were lost to follow-up due to termination of pregnancy (TOP) or neonatal death. Out of the remaining 34 fetuses with structural posterior fossa malformation, 23 agreed to participate in the VABS-II questionnaire, adding to a total of 93 fetuses with long-term neurodevelopmental outcome follow-up. These results are further described in Figure 1.

The demographics of the study population are presented in Table 2, which further compares the demographics of the fetuses with structural posterior fossa malformation with those of the apparently healthy fetuses. No statistically significant differences were found in the maternal and newborn characteristics between the two groups. Regarding pregnancy characteristics, in the posterior fossa malformations group, MRI was conducted at an earlier GA (32 weeks, IQR 31–33.5 weeks, *p* < 0.001).

**Figure 2:**
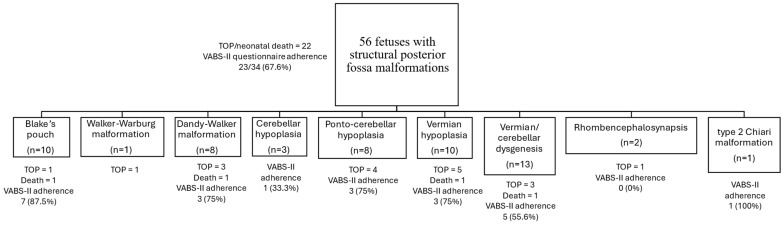
Further division of the fetuses with posterior fossa malformation by the type of malformation, VABS-II adherence, and neonatal outcome (termination of pregnancy or neonatal death). TOP—termination of pregnancy, VABS-II—Vineland Adaptive Behavior Scales II.

Figure 2 describes the type of malformation, adverse neonatal outcomes (TOP or neonatal death), and participation in the VABS-II questionnaire among the 56 fetuses with structural posterior fossa malformation included in our study. Cerebellar malformations were the most common (66.1%), while cystic malformations were fewer (32.1%), and only one fetus had cranial vault malformation (1.7%). Rates of TOP or neonatal death varied among the different malformations, with the highest rates among Walker–Warburg malformation (100%), vermian hypoplasia (60%), and Dandy–Walker malformation (50%). Participation in the VABS-II questionnaire for most of the malformations ranged from 75–87.5%, with lower participation among fetuses with cerebellar hypoplasia (33%) and rhombencephalosynapsis (0%).

Table 3 compares long-term neurodevelopmental outcomes between fetuses with structural posterior fossa malformations and apparently healthy fetuses, as reflected in the VABS-II questionnaire results. No statistically significant differences were found in the VABS-II composite score. The motor skills domain scores were lower among fetuses with structural posterior fossa malformations, though their mean score remained in the normal range (87.7 ± 16.5, *p* = 0.01). No statistically significant differences were found in any of the other three domains. Furthermore, no statistically significant difference was found when comparing the number of cases with abnormal VABS-II scores between the two groups, including in the motor skills domain.

**Figure 3:**
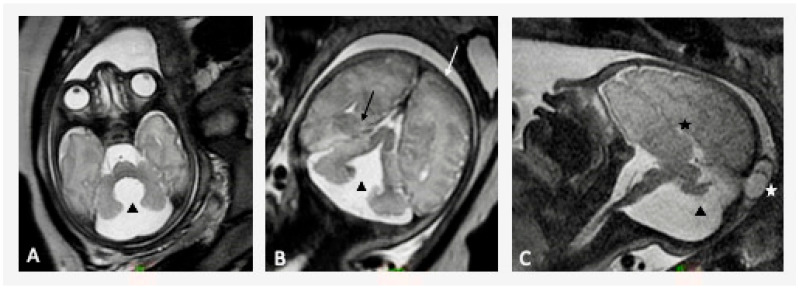
Axial (**A**), coronal (**B**), sagittal (**C**) T2-MR fetal brain images at 35 weeks of gestation. Dandy–Walker malformation (black triangle) accompanied by complete agenesis of corpus callosum (black star), occipital meningocele (white star), closed lip schizencephaly (black arrow), polymicrogyria (white arrow).

A total of four fetuses with structural posterior fossa malformations presented abnormal long-term neurodevelopmental outcomes, as reflected by the VABS-II questionnaire composite score. Table 4 describes these four cases, and Figure 3 presents MRI findings of one of these four cases. All four cases presented with additional structural CNS anomalies, and with anomalies in other systems, such as ventricular septal defects, gastroschisis, and skeletal deformities. Two cases presented chromosomal abnormalities, one with Down syndrome and one with chromosomal deficiencies in CMA, while another case was diagnosed with autism. Two cases were conceived by IVF, with one of them utilizing ICSI.

Among the 19 fetuses with structural posterior fossa malformation and normal long-term neurodevelopmental outcomes, 10 fetuses (52.6%) presented with additional CNS anomalies—5 with lateral ventricle asymmetry, 1 with lateral ventricle asymmetry combined with segmental hemangioma in the head (suspected for PHACES syndrome), 1 with corpus callosum hypoplasia, 1 with spinal meningomyelocele (which was corrected during pregnancy) with lateral ventricle enlargement (15 mm), and 1 diagnosed with VACTER syndrome after birth. Two cases (10.5%) presented with chromosomal abnormalities, both were CMA abnormalities: one was a variant of uncertain significance (VUS), and one was a known variant that may cause a developmental delay. Eight of these fetuses (42.1%) presented with additional extracranial anomalies, which included spina bifida, renal defects, cardiac defects, and facial deformities.

The motor skills domain VABS-II score was missing for 17 out of the 93 subjects who participated in the VABS-II questionnaire. Table 5 describes key characteristics of these subjects, including a comparison with subjects for whom data were gathered. The two groups differed only in median age at VABS-II completion (7.41 years with an IQR of 7.41–7.83 years for the missing data group vs. 3.2 years with an IQR of 1.93–5.43 years for the gathered data group). No statistically significant differences were found regarding the presence of posterior fossa malformations, birth weight and growth percentile, gestational age at birth, low 5-min APGAR score, or composite VABS-II score.

## 4. Discussion

Among our study population, the overall long-term neurodevelopmental outcomes of fetuses with structural posterior fossa malformation did not differ from those of apparently healthy fetuses. Among different domains of neurodevelopment, a difference was found only in the motor skills domain, in which children with structural posterior fossa malformation demonstrated lower mean VABS-II scores, but with no statistically significant difference in the number of abnormal cases, and with mean scores remaining in the normal range, implying that this finding is probably without clinical significance.

In our study, none of the fetuses with abnormal long-term neurodevelopmental outcomes and structural posterior fossa malformation had an isolated malformation. Instead, they all had additional central nervous system malformations, with some presenting conditions and syndromes like autism and Down syndrome, which are associated with poor neurodevelopmental outcomes.

This study exhibits several strengths. The current study differs from previous studies conducted to assess the outcomes of structural posterior fossa malformations observed in fetal MRI in terms of the specific malformations examined, the measured outcomes, and the consideration of additional abnormalities. Previous research published by Tilea et al. [19] did not address any postnatal outcomes, as it compared fetal MRI of cases that ended in TOP with whole fetus fetopathological examinations. A systematic review [11] by Bolduc et al. demonstrated that most studies have focused primarily on neurological impairment and IQ measurement, while neurodevelopmental outcomes, such as cognition, communication, socialization and behavior have rarely been investigated. We addressed this issue in our study by utilizing the VABS-II questionnaire, which measures neurodevelopmental outcomes in four domains, as previously described in the Materials and Methods Section.

Another strength lies in the joint analysis of several structural posterior fossa malformations. Due to the low prevalence of posterior fossa malformation, most of the current literature tends to address a specific malformation when examining neurodevelopmental outcomes. Cornips et al. [20], in their research, addressed only cases of Blake’s pouch cyst, with a series of six cases. Another study by Tarui et al. [13] investigated 20 children with isolated inferior vermian hypoplasia. In the current study, we addressed a variety of structural posterior fossa malformations observed in fetal MRI, while further comparing their neurodevelopmental outcomes with those of apparently healthy fetuses observed in fetal MRI as well. While addressing such a variety of malformations adds more variables that may affect the measured neurodevelopmental outcomes and limit the conclusions of our study, this allowed us to analyze more data, with more cases compared to previous similar studies.

We acknowledge several limitations that our study exhibits. The number of cases with posterior fossa malformation in our study was low, with 39.2% dropped due to TOP or neonatal death. This number of cases, as discussed in the previous paragraph, is common in research on posterior fossa malformations due to their low prevalence. Another limitation of our study is the utilization of telephone interviews for neurodevelopmental assessment, as this exposes the research to both interviewer bias and response bias [21,22]. This is a common limitation of studies of this type, and we addressed it by utilizing the VABS-II questionnaire, which is a reliable and validated tool for such studies. Furthermore, participation in the VABS-II questionnaire in this study was as expected considering previous studies utilizing neurodevelopmental questionnaires [18,23,24,25]. No comparison was made regarding adherence among different malformation types due to the low number of subjects in each group, but rates mostly ranged from 75% to 87.5%, as described in Figure 3. Lower participation rates were found among patients with cerebellar hypoplasia (33%) and rhombencephalosynapsis (0%), most likely due to only 3 and 2 patients presenting these specific malformations, respectively. The motor skills domain VABS-II score was missing in roughly 18% of the subjects who participated in the VABS-II questionnaire. Data regarding the cause of the missing data were not recorded during the data gathering stage. This may have led to exclusion bias. To try and mitigate the bias, post hoc analysis of the missing cases was conducted, demonstrating differences only in the age of the subjects during the VABS-II interview, which was higher among subjects with missing data. No differences in composite outcomes were observed. Another limitation is the absence of data regarding neurodevelopmental and functional outcomes in adolescence and adulthood. Further research is needed to establish the outcomes of these fetuses beyond early childhood.

## 5. Conclusions

The long-term neurodevelopmental outcomes in structural posterior fossa malformations among our study population were found to be similar to that of an apparently healthy fetus. Cases with abnormal outcomes presented severe posterior fossa malformations and additional abnormalities (structural, developmental, and/or chromosomal). This suggests that a fetus diagnosed with structural posterior fossa malformation on fetal MRI may exhibit a normal long-term neurodevelopmental outcome, considering that no additional abnormalities are detected in prenatal screening that includes proper sonographic, biochemical, and genetic screening, as well as fetal MRI. More extensive research with a wider pool of participants and with an assessment of neurodevelopmental outcomes later in life should be conducted to aid in further establishing our results and to further aid clinical decision-making.

## Figures and Tables

**Table 1:** Subject inclusion and exclusion criteria.

Inclusion Criteria	Exclusion Criteria
Fetal MRI conducted at our tertiary Medical Center between 2011 and 2019, due to the following indications: Previous child with prenatal neurologic findings; Previous abnormal pregnancy; Fetal ultrasound screening with abnormal cranial findings; Suspicion of cytomegalovirus infection, ruled out by amnio-polymerase chain reaction.	Fetal MRI for an indication other than those described in the inclusion criteria.
	Poor-quality MRI examination, preventing the production of essential information.
	Functional anomalies of the posterior fossa.
	Lack of sufficient recorded data.
	Multiple gestation.

**Table 2:** Study population characteristics.

**Variable**	**Total Population** **(*n* = 104)**	**Posterior Fossa Malformations Group** **(*n* = 34)**	**Apparently Healthy Fetuses Group** **(*n* = 70)**	***p*** **-Value**
Maternal characteristics				
Maternal age at pregnancy (years)	(*n* = 103)	(*n* = 33)		
Median (IQR)	33 (29–36)	31 (28–36)	34 (29.75–36)	0.21
Parity	(*n = 98)*	(*n* = 28)		
Median (IQR)	1 (0–2)	1.5 (0–3)	1 (0–2)	0.12
Gravida	(*n* = 98)	*(n* = 28)		
Median (IQR)	3 (1–4)	3 (1–5)	3 (1–4)	0.35
Thyroid disorders	6/98 (6.1%)	1/28 (3.6%)	5 (7.1%)	0.67
Coagulation disorders	11/98 (11.2%)	4/28 (14.3%)	7 (10%)	0.50
Anemia	4/98 (4.1%)	0/28 (0%)	4 (5.7%)	0.57
Hypertension	4/98 (4.1%)	3/28 (10.7%)	1 (1.4%)	0.07
Pregestational diabetes	2/99 (2.0%)	0/29 (0%)	2 (2.9%)	>0.99
Smoking	4/98 (4.1%)	2/28 (7.1%)	2 (2.9%)	0.32
Current pregnancy characteristics				
Type of conception	(*n* = 97)	(*n* = 28)	(*n* = 69)	
Spontaneous	83 (85.6%)	23 (82.1%)	(87.0%) 60	0.54
Fertilization treatments	14 (14.4%)	5 (17.9%)	9 (13.0%)	
Male fetal gender	54/102 (52.9%)	20/32 (62.5%)	34 (48.6%)	0.19
GA at MRI (weeks)	(*n* = 103)	(*n* = 33)	34 (32–36)	
Median (IQR)	33 (32–35)	32 (31–33.5)		<0.001 *
Ab. NT	5/89 (5.6%)	2/20 (10%)	3/69 (4.3%)	0.31
Ab. echocardiogram	10/46 (21.7%)	5/19 (26.3%)	5/27 (18.5%)	0.72
Ab. CMA	5/36 (13.9%)	4/14 (28.6%)	1/22 (4.5%)	0.06
Newborn characteristics				
Birth weight (grams)	(*n* = 94)	(*n* = 26)	(*n* = 68)	
Mean (SD)	3116 (630)	2887 (850)	3204 (503)	0.08
Growth percentile (Dolberg) at birth	(*n* = 94)	(*n* = 26)	*(n = 68)*	
Mean (SD)	51.4 (29.6)	47.35 (33.6)	52.9 (27.9)	0.41
Mode of delivery	(*n* = 95)	(*n* = 26)	(*n* = 69)	0.15
Vaginal	53 (55.8%)	12 (46.2%)	41 (59.4%)	
Cesarean	38 (40.0%)	14 (41.2%)	24 (34.8%)	
Instrumental	4 (4.2%)	0 (0%)	4 (5.8%)	
GA at delivery (weeks)	(*n* = 95)	(*n* = 26)	(*n* = 69)	0.33
Median (IQR)	38 (37–39)	38.5 (36–39)	38 (38–39)	
Apgar score under 8 (at 5 min)	1/89 (1.1%)	1/20 (5%)	0/69 (0%)	0.23

Ab.—abnormal, CMA—chromosomal microarray, GA—gestational age, IQR—interquartile range, MRI—magnetic resonance imaging, NT—nuchal translucency, SD—standard deviation. * Statistically significant result.

**Table 3:** Comparison of long-term neurodevelopmental outcomes.

	**Total Population** **(*n* = 93) **	**Posterior Fossa Malformations Group** **(*n* = 23) **	**Apparently Healthy Fetuses Group** **(*n* = 70)**	***p*** **-Value**
VABS-II Adherence	- †	23/34 (67.6%)	- †	-
Age at VABS-II (years) Median (IQR)	4.33 (2.0–6.2)	3.75 (1.7–5.1)	4.63 (2.2–6.3)	0.21
Composite outcome Mean (SD)	98.2 (15.4)	93.4 (19.0)	99.8 (13.8)	0.08
Abnormal (score < 85)	13 (14.0%)	4 (17.4%)	9 (12.9%)	0.73
Communication Mean (SD)	102.8 (17.9)	96.6 (20.6)	104.8 (16.6)	0.06
Abnormal (score < 85)	14 (15.1%)	6 (26.1%)	8 (11.4%)	0.10
Daily living skills Mean (SD)	98.7 (15.4)	96.1 (20.3)	99.6 (13.5)	0.34
Abnormal (score < 85)	12 (12.9%)	4 (17.4%)	8 (11.4%)	0.48
Socialization Mean (SD)	96.3 (13.6)	94.6 (18.8)	96.9 (11.5)	0.58
Abnormal (score < 85)	13 (14.0%)	5 (21.7%)	8 (11.4%)	0.29
Motor skills Mean (SD)	(*n* = 76) 96.4 (17.7)	(*n* = 19) 87.7 (16.5)	(*n* = 57) 99.3 (17.2)	0.01 *
Abnormal (score < 85)	17 (22.4%)	7 (36.8%)	10 (17.5%)	0.11
Abnormality (score < 85) in any domain	25/76 (32.9%)	8/19 (42.1%)	17/57 (29.8%)	0.32

Neurodevelopmental scores above were measured using the VABS-II questionnaire. SD—standard deviation. * Statistically significant result. † Adherence within these groups could not be assessed due to the study recruitment process, as discussed in the Materials and Methods Section.

**Table 4:** Detailed description of the four fetuses with posterior fossa malformation and abnormal long-term neurodevelopmental outcomes.

**Gender**	**GA at Birth**	**Type of Malformation**	**Malformation Description**	**Additional Structural Anomalies**	**Genetic or Neurodevelopmental Anomalies**	**Age While Participating in VABS-II (Years)**	**VABS-II Composite Score**	**Neurodevelopmental Percentile (%)**
Male	38 + 5	Vermian hypoplasia		Closed lip schizencephaly in the left frontal lobe, microcephaly, oligohydramnios, ventricular septal defect	Deficiencies in chromosomes 10 and 13 in CMA	6.75	54	<1
Male	36 + 6	Dandy–Walker malformation	Enlarged posterior fossa, upward displacement of the tentorium and the torcular, vermian agenesis, hypoplastic brain stem	Complete agenesis of CC, occipital meningocele, closed lip schizencephaly, polymicrogyria		7.08	55	<1
Male	34 + 1	Blake’s pouch		Lateral ventricles asymmetry (7 mm, 19 mm), gastroschisis	Autism	2.33	64	1
Female	37 + 6	Cerebellar dysgenesis	Cerebellar right lobe is hypoplastic and irregular with porencephalic cyst	Short long bones, absence of the nasal bone, curly toe in the left foot	Down syndrome	1	70	2

GA—gestational age.

**Table 5:** Description of cases missing VABS-II motor skills domain score.

**Variable**	**Missing Motor Skills Domain Score** **(*n* = 17) **	**Motor Skills Domain Score Known** **(*n* = 76) **	***p*-Value**
Posterior fossa malformation	4 (30.7%)	19 (25%)	>0.99
Birth weight (grams)			
Median (IQR)	3090 (2674–3825)	3140 (2808–3483)	0.80
Growth percentile (Dolberg) at birth Median (IQR)	48 (29–85)	50 (25–79.75)	0.89
GA at delivery (weeks) Median (IQR)	38 (36.25–39.75)	38.5 (38–39)	0.43
Apgar score under 8 (at 5 min)	0/16 (0%)	1/70 (14.2%)	
Age at VABS-II (years)			>0.99
Median (IQR)	7.41 (7.41–7.83)	3.20 (1.93–5.43)	<0.001 *
Composite outcome Median (IQR)	102 (87.5–114.5)	98 (89.25–109)	0.68
Abnormal (score < 85)	3 (21.4%)	10 (13.1%)	0.70

GA—gestational age, IQR—interquartile range, MRI—magnetic resonance imaging, NT—nuchal translucency. * Statistically significant result.

## Data Availability

The data presented in this study are available upon request from the corresponding author due to patient privacy restrictions.

## Abbreviations

The following abbreviations are used in this manuscript:

CMA	Chromosomal microarray
DWM	Dandy–Walker malformation
FOV	Field of view
GA	Gestational age
MCM	Mega cisterna magna
MRI	Magnetic resonance imaging
TOP	Termination of pregnancy
US	Ultrasonography
VABS-II	Vineland Adaptive Behavior Scales II
VSD	Ventricular septal defect
VUS	Variant with uncertain significance
WWS	Walker–Warburg syndrome
